# Evaluation of Anesthetic and Cardiorespiratory Effects after Intramuscular Administration of Three Different Doses of Telazol^®^ in Common Marmosets (*Callithrix jacchus*)

**DOI:** 10.3390/vetsci10020116

**Published:** 2023-02-03

**Authors:** Anna Goodroe, Jaco Bakker, Edmond J. Remarque, Corinna N. Ross, Diana Scorpio

**Affiliations:** 1 Veterinary Services, Southwest National Primate Research Center, Texas Biomedical Research Institute, San Antonio, TX 78227, USA; 2 Animal Science Department, Biomedical Primate Research Centre, 2288 GJ Rijswijk, The Netherlands; 3 Virology Department, Biomedical Primate Research Centre, 2288 GJ Rijswijk, The Netherlands; 4 Research Services, Southwest National Primate Research Center, Texas Biomedical Research Institute, San Antonio, TX 78227, USA

**Keywords:** anesthesia, common marmoset, immobilization, recovery period, sedation, Telazol^®^, tiletamine, zolazepam

## Abstract

**Simple Summary:**

Our study demonstrates that the immobilization of marmosets is best performed with a Telazol^®^ dose of 5 mg/kg administered intramuscularly. This dose resulted in 50–90 min of immobilization, during which scientists and veterinarians can perform minimally invasive procedures.

**Abstract:**

Marmosets’ small body size makes anesthesia challenging. Ideally, small volumes of drugs should be administered intramuscularly (i.m.). In addition, dose-dependent sedation and anesthesia are desirable properties for sedatives and anesthetics in marmosets. Telazol^®^ (tiletamine and zolazepam) is highly concentrated, allowing the use of small injection volumes and dose-dependent sedation and anesthesia. A randomized, blinded study with crossover design in ten healthy adult common marmosets (*Callithrix jacchus*) was performed to evaluate the anesthetic and cardiorespiratory effects of three doses of i.m. Telazol^®^ (respectively, 5, 10, and 15 mg/kg). Depth of anesthesia, cardiorespiratory effects, and induction, immobilization, and recovery times were determined. A significant difference was observed in immobilization time between 5 and 15 mg/kg of Telazol^®^. In addition, 15 mg/kg of Telazol^®^ resulted in increased recovery times compared to 5 mg/kg. The cardiorespiratory effects during the first 45 min of immobilization were within clinically acceptable limits. The pedal withdrawal reflex was the best indicator of the anesthetic depth.

## 1. Introduction

Common marmosets (*Callithrix jacchus*) are frequently used as biomedical research models, e.g., for neuroscience, aging, metabolic disorders, behavioral, and infectious diseases studies [1]. The marmoset’s relative small body size (an adult marmoset weighs 350–450 g) allows alert handling [1]. Marmosets can be acclimated to participate in many research procedures avoiding the use of sedation or anesthesia. Sedation results in central nervous system depression and relaxation, but the animal will respond to noxious stimuli. Anesthesia results in loss of sensation in all or parts of the body, with no response to noxious stimuli [2]. Sedatives and anesthetics are preferably administered by intramuscular (i.m.) injection, which facilitates their quick absorption into the bloodstream and therefore results in a short induction time. However, the small muscle mass of marmosets limits the volume of drug that can be administered via i.m. injection [3]. Currently, there are few publications presenting evidence-based sedation and anesthetic injectable drug doses for marmosets [4,5,6,7,8,9,10]. An ideal injectable sedative or anesthetic drug for use in marmosets would allow a small injection volume and provide muscle relaxation, and its effect would be dose-dependent to facilitate either sedation or anesthesia. Telazol^®^ (Zoetis; Kalamazoo, MI, USA) has those properties. 

Telazol^®^ is a combination of tiletamine and zolazepam in a 100 mg/mL concentration (50 mg/mL tiletamine and 50 mg/mL zolazepam). Tiletamine is a nonnarcotic, nonbarbiturate, injectable dissociative anesthetic agent. Zolazepam is a benzodiazepine with minor tranquilizing properties. Their combination provides significant muscle relaxation [2]. The safe use of Telazol^®^ has already been documented in nonhuman primates including capuchin monkeys [11,12], owl monkeys [13], rhesus macaques [14,15,16], cynomolgus macaques [17], baboons [14], chimpanzees [18,19,20], and southern brown howler monkeys [21]. There are a few reports of Telazol^®^ administration to marmosets, showing a wide dose range [9,22,23,24,25,26,27,28]. However, evidence-based sedation and anesthetic dose determination for marmosets have never been performed.

We hypothesized that there would be a dose-dependent effect of Telazol^®^ in marmosets offering the opportunity to induce sedation (low dose of Telazol^®^) or anesthesia (high dose of Telazol^®^). We predicted that a high dose of Telazol^®^ administered i.m. would elicit a longer immobilization period and a longer time to the return of the positive pedal withdrawal reflex (noxious response evaluation) than lower doses. In order to evaluate the depth and length of sedation or anesthesia, we characterized the cardiorespiratory effects and the induction, immobilization, and recovery times elicited by 5, 10, or 15 mg/kg of Telazol^®^ administered i.m. to marmosets. 

## 2. Materials and Methods

### 2.1. Animals, Housing, and Husbandry

Ten healthy adult common marmosets (*Callithrix jacchus*) housed at the Southwest National Primate Research Center (SNPRC, San Antonio, TX, USA) were included in this study (five females and five males). The body weight of the included animals was 412 ± 43 g, and their age was 4.0 ± 1.1 years. The marmosets were maintained using the standardized husbandry protocols at SNPRC [29]. The facilities were maintained at 74 to 90 °F ‘(23.3 to 32.2 °C)’ and humidity of 30 to 70%. The marmosets were socially housed and fed a base diet consisting of Mazuri (5LK6) or Teklad (TD.130059.PWD) with additional enrichment food items provided daily. Water was continuously available via water bottles. Husbandry consisted in the daily removal of debris from the enclosures and a biweekly sanitation of the enclosures. The marmosets were provided structural and manipulable enrichment within the enclosures.

All procedures were approved by the Institutional Animal Care and Use Committee of the Texas Biomedical Research Institute. Animal care and use were consistent with the ASP Principles for Ethical Treatment of Non-Human Primates and compliant with the US National Research Council’s Guide for the Care and Use of Laboratory Animals, the US Public Health Service’s Policy on Humane Care and Use of Laboratory Animals, and USDA Animal Welfare Act and Regulations.

### 2.2. Study Method and Data Analysis 

A prospective, randomized, blinded, crossover design was used (with each animal serving as its own control). Two groups of five animals each were formed with a random number generator with sex accounted for, to ensure an equal distribution of males and females. One group received i.m. Telazol^®^ 5 mg/kg, followed by 10 mg/kg and then 15 mg/kg, and the second group received i.m. Telazol^®^ 15 mg/kg, followed by 10 mg/kg and then 5 mg/kg. There was at least a 28-day period between Telazol^®^ administration time points to ensure the elimination of Telazol^®^ (the terminal elimination period phase was 8.4 h in pigs [30]).

On the day of Telazol^®^ administration, the marmosets were not fed in the morning. For the injection, one person manually restrained the animal, while a second person administered Telazol^®^ into the left or right quadriceps muscle, using a syringe with a permanently attached 26-gauge needle. Care was taken that the drugs were not injected directly into the bloodstream. The total volume injected ranged from 0.02 to 0.07 mL. Subsequently, the marmoset was returned to its nest box. Once the marmoset exhibited muscle relaxation and did not maintain a normal upright posture, it was removed and placed on a thermal support blanket (Bair Hugger, 3M, Eden Prairie, MN, USA). The rectal temperature was continuously monitored, and if it reached 101 °F (38.3 °C), thermal support was discontinued until the temperature reached 99 °F (37.2 °C) or lower. The thermal support blanket setting was adjusted to maintain the desired rectal temperature range of 99–101 °F (37.2–38.3 °C).

The effects of Telazol^®^ were evaluated by a treatment-blinded observer. The following time points were recorded: time from injection until loss of righting reflex (induction time), time from loss of righting reflex to demonstrating intentional spontaneous movement (immobilization time), time from intentional spontaneous movement to time when the marmoset could safely walk and climb within the enclosure (recovery time). A loss of the righting reflex was defined as the inability to regain the normal upright posture when placed in lateral recumbency.

Once the marmoset exhibited sufficient muscle relaxation, a pulse oximeter monitor clip (Leading Edge Veterinary Equipment, Centennial, CO, USA) was placed on a limb to obtain heart rate and hemoglobin oxygen saturation (SpO_2_%). A thermometer (Welch Allyn, Milwaukee, WI, USA) was inserted in the rectum, allowing for continuous temperature monitoring. A blood pressure cuff (HDO Non-invasive Blood Pressure System, S + B medVet Systeme and Beratung, Bahenhausen, Germany) to indirectly measure the blood pressure was placed around a hindlimb. The physiological parameters were measured every five minutes until spontaneous movement prevented the monitoring probe attachment. Respiratory rate (number of breaths per minute) was assessed by visual observation of the chest movement over a 30 s period and multiplied by two. 

Posture was assessed every five minutes post administration of Telazol^®^ until the animal was fully recovered. During the immobilization period, the following parameters were assessed to determine the depth of sedation or anesthesia: muscular tension, pedal withdrawal reflex, and palpebral reflex (Table 1). The scoring system was derived from previous injectable drug evaluation studies in marmosets [4,5,31]. Posture was assessed by visual observation of the marmoset. Muscular tension was assessed by flexing and extending the marmoset’s limbs. Pedal withdrawal was assessed by applying a tissue forceps for one second on the second digit of the left rear limb immediately below the nail. The palpebral reflex was assessed by lightly touching the medial canthus (not the cornea) of the left eye with a dry cotton swab. 

Once the marmosets exhibited intentional spontaneous movement, they were placed into a clear plastic box on top of a thermal support blanket. Once the marmosets exhibited a stable posture and balance, they were placed in a mesh enclosure like their home enclosure. Once they were able to safely ambulate throughout the enclosure, they were considered recovered and reunited with their companions in their home enclosure. 

Statistical tests were performed with R studio v4.1.3. The data are presented as mean ± SD or as mean difference with 95% confidence intervals. To determine the statistical significance of the differences in the induction, immobilization, and recovery times, mixed models were performed (lme4 R and emmeans packages). The model employed was the following: Time ~ N(β_0_, γ_animal_) + (β_dose_ * dose [5,10,15]) + N(0, σ), where Time is the induction, immobilization, recovery, or total time; N represents a normal distribution with expectation and standard deviation, respectively; β_0_ is the regression intercept; γ_animal_ is the between-subject variability; β_dose_ is the coefficient representing the effects of dose, and σ is the standard deviation of the residual error. The time estimates for the three doses with corresponding 95% confidence intervals were obtained using the emmeans package. The *p* values were not adjusted for multiple comparisons.

## 3. Results

**Figure 1:**
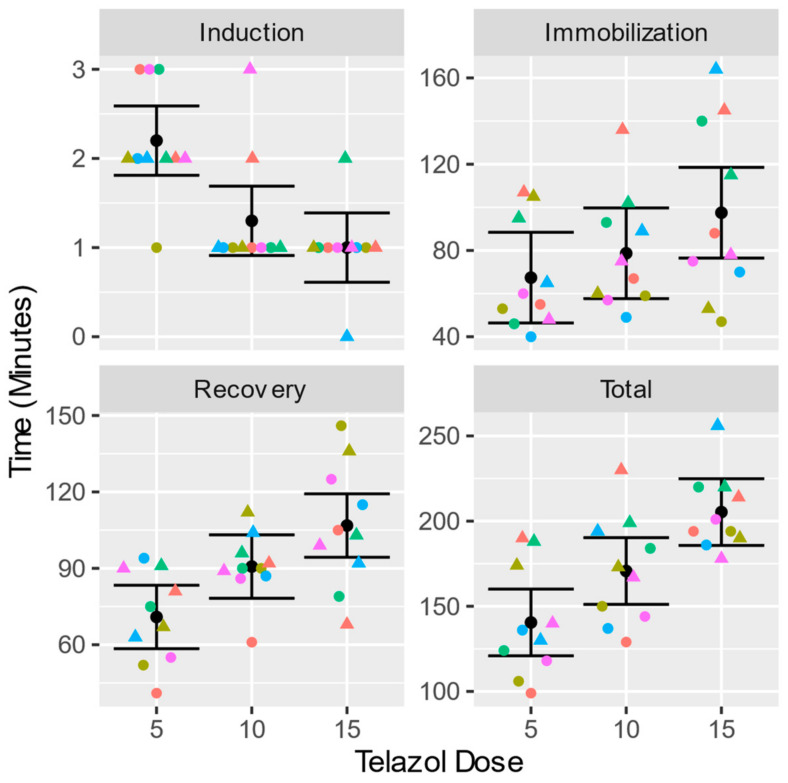
Induction, immobilization, recovery, and total times with three different doses of Telazol^®^. The error bars show the marginal means and 95% confidence intervals. The individual times are plotted as circles for animals receiving 5, 10, and 15 mg/kg of Telazol^®^ and as triangles for animals receiving 15, 10 and 5 mg/kg. Individual animals are represented by the symbols’ shape and color.

The induction time was shorter for the 15 and 10 mg/kg doses compared to 5 mg/kg dose (Figure 1, 5 vs. 10: 0.9 (0.3 to 1.5) *p* = 0.0035, 5 vs. 15: 1.2 (0.6 to 1.8) *p* = 0.00029, 10 vs. 15: 0.3 (−0.3 to 0.9) *p* = 0.515). The mean time to produce immobilization was 1.00 (0.61 to 1.39) min for the 15 mg/kg dose, 1.3 (0.91 to 1.69) min for the 10 mg/kg dose, and 2.20 (1.81 to 2.59) min for the 5 mg/kg dose.

There was a difference in the length of immobilization produced by 5 and 15 mg/kg of Telazol^®^ (Figure 1, 5 vs. 10: −11.3 (−32.9 to 10.3) *p* = 0.287, 5 vs. 15: −30.1 (−51.7 to −8.5) *p* = 0.00911, 10 vs. 15: −18.8 (−40.4 to 2.8) *p* = 0.085). The mean immobilization time was 97.5 (76.5 to 119) min for the 15 mg/kg dose, 78.7 (57.7 to 99.7) min for the 10 mg/kg dose, and 67.4 (46.4 to 88.4) min for the 5 mg/kg dose. There was variability in the immobilization times elicited by the 5 mg/kg dose within a range from 40 to 107 min. A similar range and variability were found for the 10 mg/kg dose (from 9 to 136 min) and the 15 mg/kg dose (from 47 to 164 min).

The recovery time after the 5 mg/kg dose was shorter than after the 10 or 15 mg/kg doses, while there was no statistically significant difference between the recovery times after the administration of the 10 and the 15 mg/kg doses (Figure 1, 5 vs. 10: −19.8 (−37.8 to −1.8) *p* = 0.033; 5 vs. 15: −35.9 (−53.9 to −17.9) *p* = 0.00056, 10 vs. 15: −16.1 (−34.1 to 1.9) *p* = 0.077). The average recovery time was 107 (94.3 to 119) min for the 15 mg/kg dose, 90.7 (78.2 to 103) min for the 10 mg/kg dose, and 70.9 (58.4 to 83.4) min for the 5 mg/kg dose. The recovery time ranged from 41 to 94 min for the 5 mg/kg dose, from 61 to 112 min for the 10 mg/kg dose, and from 68 to 146 min for the 15 mg/kg dose.

The total procedure time for the 5, 10, and 15 mg/kg doses differed (Figure 1, 5 vs. 10: −30.2 (−49.7 to −10.7) *p* = 0.0044, 5 vs. 15: −64.8 (−84.3 to −45.3) *p* = 0.0000016, 10 vs. 15: −34.6 (−54.1 to −15.1) *p* = 0.0015). The average total procedure time was 205.3 (186 to 225) min for the 15 mg/kg dose, 171 (151 to 190) min for the 10 mg/kg dose, and 140 (121 to 160) min for the 5 mg/kg dose. Table 2 provides the statistical summary of our results.

Table 3 provides the main results of subjective assessments of sedation or anesthetic depth. Details are provided in Figure S1. The physiological data of the first 45 min of immobilization are shown in Table S1. The monitored parameters were within clinically acceptable limits during all protocols, and there were no clinical concerns requiring intervention. Respiratory rate and heart rate generally decreased post injection of Telazol^®^. The SpO_2_ % values were consistently low across all Telazol^®^ doses, but no cyanosis was observed.

The Group 1 (order of dose administration 5, 10, 15 mg/kg) and Group 2 (order of dose administration 15, 10, 5 mg/kg) induction, immobilization, and recovery procedure times were compared. There was a statistically significant difference (*p* = 0.00344) between Group 1 and 2 for total time at a dose of 5 mg/kg (Group 1 mean 116.6 (95.3 to 137.9) min and Group 2 mean 164.4 (134.3 to 194.5) min).

## 4. Discussion

This study evaluated the length and characters of the induction, immobilization, and recovery times, anesthetic depth, and effect on cardiorespiratory parameters of Telazol^®^ administered i.m. in marmosets. It showed that 5 mg/kg is the advised dose for common procedures such as blood draws or minor brief surgeries. This dose results in approximately 50 to 90 min of immobilization.

The high doses of Telazol^®^ (10 and 15 mg/kg) resulted in decreased induction times compared to the 5 mg/kg dose, but only the 15 mg/kg dose provided a longer immobilization time compared to the 5 mg/kg dose. There was a significant increase in the immobilization time with 15 mg/kg of Telazol^®^, producing approximately 80 to 120 min of immobilization. The marmosets sedated with 15 mg/kg Telazol^®^ required a longer period of time before the return of the pedal withdrawal reflex, normal muscle tension, and spontaneous movement than the marmosets sedated with the 5 mg/kg dose (Table 3). It is worth noting the wide range of these time points, reflecting variance in the length of sedation or surgical general anesthesia produced by Telazol^®^ at both doses.

The immobilization time determines the number and type of procedures that can be completed. The recovery time for the 5 mg/kg dose (approximately 60 to 80 min) was significantly shorter than that of the 15 mg/kg dose (approximately 90 to 120 min). The authors suggest that a longer immobilization period may not be advantageous because of a prolonged recovery period. Prolonged recovery periods have also been documented in wolves [32] and crested porcupines [33]. The recovery time may be impacted by species variance in the relative rates of metabolism of tiletamine and zolazepam [2].

It is critical to match a procedure length and potential to elicit pain to appropriate sedation or anesthesia protocols. If the anesthetic event is too long, the animal is at risk of hypothermia due to prolonged immobilization, and the return to normal activity and behavior is delayed. It is advantageous to have a well-characterized injectable drug regimen to match the procedure length to the drug dose. Optimal recoveries return an animal to normal physiological function as quickly as possible. Doses of 5–15 mg/kg of Telazol^®^ were sufficient to produce a surgical plane of anesthesia for very short invasive procedures lasting less than 10 min. The muscle relaxation elicited by Telazol^®^ will be helpful for invasive procedures (trauma repairs, biopsies, etc.). Doses of 5–15 mg/kg of Telazol^®^ produced a sedation event sufficient for non-invasive procedures such as imaging. The range of the immobilization periods reflects differences in the response to Telazol^®^ in individual animals. The observed difference between Group 1 and 2 in immobilization time at the 5 mg/kg dose is most likely to reflect the variance in individual responses to Telazol^®^.

No marmoset required clinical intervention during the administration of Telazol^®^, and no clinical adverse effects were documented for the doses administered. Thermal support was provided, as the authors experience has found that hypothermia with any prolonged sedation event is common in marmosets [4,5]. One should keep in mind that the environmental temperature is a significant variable that may increase or decrease the marmoset’s core temperature, resulting in potential changes in blood pressure and drug metabolism. External heat support is recommended for all sedation protocols in marmosets. Cynomolgus macaques exhibited a lower body temperature when sedated with Telazol^®^ compared to ketamine [17]. The heart rate was noted to decrease over time, especially from 5 to 15 min post injection (Table S1). The heart rate values reported by the blood pressure machine were lower than those reported by the SpO_2_ monitor; this difference was not clinically significant, and the authors suggest using one monitor to evaluate the changes in heart rate over time. There were no variations in systolic, diastolic, and mean blood pressure between the doses nor over time post Telazol^®^ administration (Table S1). The lack of clinically significant cardiac changes correlates with previous Telazol^®^ evaluations in capuchins [11] and owl monkeys [13]. The respiratory rate decreased over time for all doses (Table S1). The SpO_2_% values (Table S1) were consistently low across all Telazol^®^ doses, and the values did not reflect the clinical appearance of the animals (no cyanosis was observed). The values were likely low due to the equipment poor signal detection rather than to poor oxygenation, based on the observed normal heart rate, respiratory rate, and no visible cyanosis. Based on these findings, it is recommended at a minimum to monitor heart rate and respiratory rate when administering Telazol^®^ to marmosets.

A positive palpebral reflex was noted approximately 8 min after injection for all three doses (Table 3). The Telazol^®^ package insert indicates that this reflex may not be diminished. Our data suggest that the palpebral reflex is not a reliable indicator of the depth of anesthesia in marmosets. The pedal withdrawal reflex is typically lost after the palpebral reflex, since it is associated with the response to noxious stimuli. This reflex was not present until 9.5 to 22 min after injection, but the standard deviation for the return of this reflex amongst the doses was high, suggesting a marked individual variation of the anesthetic depth (Table 3). While the immobilization time was not significantly different between the three doses, the longer length of time before a positive pedal withdrawal reflex at higher doses of Telazol^®^ suggests that the overall depth of anesthesia was greater during the immobilization period. Normal muscle tension returned, on average, after a positive pedal withdrawal reflex (Table 3). We recommend relying on the pedal withdrawal reflex as a better indicator of the current anesthesia depth.

Besides the pedal withdrawal reflex, no other noxious stimulus was implemented to evaluate the appropriate anesthesia depth. Individual variation in the pedal withdraw reflex was significant, and a larger sample size may have further refined the dose characterization.

We have provided an evidence-based drug regimen for clinical and research use in the marmoset community. Safe, effective sedation and anesthetic protocols have a major influence on the welfare of laboratory animals. Improvement in anesthesia delivery and outcomes is an essential refinement, as anesthesia can have serious adverse effects on the quality of the results obtained from animal studies. The low volume of this injectable drug is a refinement, given the larger volumes required for commonly used drugs such as ketamine and alphaxalone [4,5].

## 5. Conclusions

Our study showed a significant increase in the immobilization and recovery periods when marmosets were administered 15 mg/kg of Telazol^®^. There was a longer period of time before a positive pedal withdrawal response for higher doses of Telazol^®^, but the variance between the animals reflected inconsistent depth and length of anesthesia elicited by Telazol^®^ in marmosets. We recommend the use of Telazol^®^ for minimally invasive procedures for this reason. We recommend a Telazol^®^ dose of 5 mg/kg administered i.m. to provide approximately 50–90 min of immobilization, given the extended recovery period with higher doses. A pedal withdrawal reflex is the recommended reflex to monitor the depth of anesthesia in marmosets under Telazol^®^ sedation.

## Figures and Tables

**Table 1:** Objective scoring system for each assessment to determine the depth of the sedation or anesthesia.

Posture	
Score	Assessment
0	Recumbency without spontaneous movement
1	Recumbency with spontaneous movement of the head or limbs
2	Sitting or standing, unstable
3	Sitting or standing, stable
Muscular Tension	
Score	Assessment
0	Complete muscle relaxation, all limbs have no muscle contraction
1	Partial muscle relaxation, one or more limbs have muscle contraction
2	Normal muscle tension, all limbs have muscle contraction
Pedal Withdrawal Reflex	
Score	Assessment
0	No reflex; no bending of the knee during or one second after the removal of the hemostat
1	Normal reflex; bending of the knee in response
2	Increased reflex; increased muscle tension and bending of the knee with muscle movements of the other limbs
Palpebral Reflex	
Score	Assessment
0	No reflex; no response of the eye lid
1	Decreased reflex; partial closing of the eye lid
2	Normal reflex: eye lid immediately closes fully

**Table 2:** Statistical summary.

Outcome	Dose Comparison	Mean Difference	Lower CL	Upper CL	*p* Value
Induction	5–10	0.9	0.34	1.46	0.00351
Induction	5–15	1.2	0.64	1.76	0.00029
Induction	10–15	0.3	−0.26	0.86	0.515
Immobilization	5–10	−11.3	−32.94	10.34	0.287
Immobilization	5–15	−30.1	−51.74	−8.46	0.0091
Immobilization	10–15	−18.8	−40.44	2.84	0.085
Recovery	5–10	−19.8	−37.84	−1.76	0.033
Recovery	5–15	−35.9	−53.94	−17.86	0.00056
Recovery	10–15	−16.1	−34.14	1.94	0.077
Total	5–10	−30.2	−49.67	−10.73	0.0044
Total	5–15	−64.8	−84.27	−45.33	0.0000016
Total	10–15	−34.6	−54.07	−15.13	0.0015

**Table 3:** Results of the assessments to determine the depth of sedation or anesthesia.

	5 mg/kg Telazol^®^	10 mg/kg Telazol^®^	15 mg/kg Telazol^®^
Spontaneous movement	37.7 min after injection	52 min	60.7 min
Normal muscle tension (score of 2)	28.5 ± 18.46 min (median) after injection	49 ± 29.49 min	52.5 ± 33.17 min
Pedal withdrawal reflex (score of 1)	9.5 ± 10.07 min after injection	10.5 ± 15.15 min	22 ± 14.39 min
Palpebral reflex (score of 1 or 2)	8 ± 4.82 min after injection	7.5 ± 1.89 min	8 ± 2.4 min

## Data Availability

Data are available on request.

## Supplementary Materials

The following supporting information can be downloaded at: https://www.mdpi.com/article/10.3390/vetsci10020116/s1, Figure S1: Results of the objective scoring for each assessment used to determine the depth of the sedation or anesthesia event; Table S1: Median and standard deviation of the physiological data collected during the first 45 min of sedation or anesthesia events.

## References

[1] Marini R.P., Wachtman L.M., Tardif S.D., Mansfield K., Fox J.G., American College of Laboratory Animal Medicine (2019). The Common Marmoset in Captivity and Biomedical Research.

[2] Grimm K.A., Lamont L.A., Tranquilli W.J., Greene S.A., Robertson S.A. (2015). Veterinary Anesthesia and Analgesia.

[3] Turner P.V., Brabb T., Pekow C., Vasbinder M.A. (2011). Administration of substances to laboratory animals: Routes of administration and factors to consider. J. Am. Assoc. Lab. Anim. Sci..

[4] Bakker J., Roubos S., Remarque E.J., Arndt S.S., Kronen P.W., Langermans J.A. (2018). Effects of buprenorphine, butorphanol or tramadol premedication on anaesthetic induction with alfaxalone in common marmosets (*Callithrix jacchus*). Vet. Anaesth. Analg..

[5] Bakker J., Uilenreef J.J., Pelt E.R., Brok H.P., Remarque E.J., Langermans J.A. (2013). Comparison of three different sedative-anaesthetic protocols (ketamine, ketamine-medetomidine and alphaxalone) in common marmosets (*Callithrix jacchus*). BMC Vet. Res..

[6] National Academies of Sciences, Engineering, and Medicine (2019). Care, Use, and Welfare of Marmosets as Animal Models for Gene Editing–Based Biomedical Research: Proceedings of a Workshop, Washington, DC, USA, 22–23 October 2018.

[7] Ishibashi H. (2016). More effective induction of anesthesia using midazolam-butorphanol-ketamine-sevoflurane compared with ketamine-sevoflurane in the common marmoset monkey (*Callithrix jacchus*). J. Vet. Med. Sci..

[8] Ludlage E., Mansfield K. (2003). Clinical care and diseases of the common marmoset (*Callithrix jacchus*). Comp. Med..

[9] Wolfe-Coote S. (2005). The Laboratory Primate.

[10] Tardif S., Bales K., Williams L., Moeller E.L., Abbott D., Schultz-Darken N., Mendoza S., Mason W., Bourgeois S., Ruiz J. (2006). Preparing New World monkeys for laboratory research. ILAR J..

[11] De La Salles A.Y.F., Andrade J.K., Lemos K.K.A., Carreiro A.D.N., de Souza J.G., Costa T.S.F., Reinaldo M., de Souza A.P., de Menezes D.J.A. (2019). Electrocardiographic parameters of *Sapajus libidinosus* (SPIX, 1823) after chemical immobilization with tiletamine-zolazepam. J. Med. Primatol..

[12] Raposo A.C., Ofri R., Schaffer D.P., Gomes Júnior D.C., Libório F.A., Martins Filho E.F., Oriá A.P. (2015). Evaluation of ophthalmic and hemodynamic parameters in capuchin monkeys (*Sapajus* sp.) submitted to dissociative anesthetic protocols. J. Med. Primatol..

[13] Chaves R.H., Souza N.F., Imbeloni A.A., Neves A.C., Teixeira R.K., Santos Cde C. (2015). Evaluation of blood pressure in feline night monkeys (*Aotus azarae infulatus*) under different restraint protocols. J. Med. Primatol..

[14] Bentson K.L., Capitanio J.P., Mendoza S.P. (2003). Cortisol responses to immobilization with Telazol^®^ or ketamine in baboons (Papio cynocephalus/anubis) and rhesus macaques (*Macaca mulatta*). J. Med. Primatol..

[15] Hernández-Godínez B., Bonilla Jaime H., Poblano A., Arteaga-Silva M., Medina Hernández A., Contreras-Uribe A., Ibáñez-Contreras A. (2019). Effect of different anesthetic mixtures-ketamine-xylazine, ketamine-acepromazine and tiletamine-zolazepam-on the physiological and blood biochemistry parameters of male rhesus monkeys. Animal Model Exp. Med..

[16] Young J.T., Vlasova R.M., Howell B.R., Knickmeyer R.C., Morin E., Kuitchoua K.I., Lubach G.R., Noel J., Hu X., Shi Y. (2021). General anaesthesia during infancy reduces white matter micro-organisation in developing rhesus monkeys. Br. J. Anaesth..

[17] López K.R., Gibbs P.H., Reed D.S. (2002). A comparison of body temperature changes due to the administration of ketamine-acepromazine and tiletamine-zolazepam anesthetics in cynomolgus macaques. Contemp. Top. Lab. Anim. Sci..

[18] Drane A.L., Calvi T., Feltrer Y., Curry B.A., Tremblay J.C., Milnes E.L., Stöhr E.J., Howatson G., Oxborough D., Stembridge M. (2021). The influence of anesthesia with and without medetomidine on cardiac structure and function in sanctuary captive chimpanzees (*Pan troglodytes*). J. Zoo Wildl. Med..

[19] Milnes E.L., Calvi T., Feltrer Y., Drane A.L., Howatson G., Shave R.E., Curry B.A., Tremblay J.C., Williams D.L. (2020). Factors Affecting Tear Production and Intraocular Pressure in Anesthetized Chimpanzees (Pan Troglodytes). J. Zoo Wildl. Med..

[20] Strong V., Moller T., Tillman A.S., Traff S., Guevara L., Martin M., Redrobe S., White K. (2018). A clinical study to evaluate the cardiopulmonary characteristics of two different anaesthetic protocols for immobilization of healthy chimpanzees (*Pan troglodytes*). Vet. Anaesth. Analg..

[21] Gonçalves G.H.P., de Souza Junior J.C., Pitz H.D.S., Peruchi A.R., Branco F.S., Hirano Z.M.B. (2019). Hematological and serum biochemistry data on southern brown howler monkeys (*Alouatta guariba clamitans*) in captivity in Brazil. J. Med. Primatol..

[22] Murphy K.L., Baxter M.G., Flecknell P.A., Abee C.R., Mansfield K., Tardif S., Morris T. (2012). Anesthesia and analgesia in nonhuman primates. Nonhuman Primates in Biomedical Research. Volume I: Biology and, Management.

[23] Burman K.J., Palmer S.M., Gamberini M., Spitzer M.W., Rosa M.G. (2008). Anatomical and physiological definition of the motor cortex of the marmoset monkey. J. Comp. Neurol..

[24] Goodroe A., Fitz C., Bakker J. (2021). Current Topics in Marmoset Anesthesia and Analgesia. ILAR J..

[25] Hahn A. (2019). Zoo and Wild Mammal Formulary.

[26] Mucker E.M., Wollen-Roberts S.E., Kimmel A., Shamblin J., Sampey D., Hooper J.W. (2018). Intranasal monkeypox marmoset model: Prophylactic antibody treatment provides benefit against severe monkeypox virus disease. PLoS Negl. Trop. Dis..

[27] Murray S. (1999). Callitrichid Husbandry Manual.

[28] Pacheco B., Menéndez-Arias L., Sodroski J. (2016). Characterization of two distinct early post-entry blocks to HIV-1 in common marmoset lymphocytes. Sci. Rep..

[29] Layne D.G., Power R.A. (2003). Husbandry, handling, and nutrition for marmosets. Comp. Med..

[30] Kumar A., Mann H.J., Remmel R.P. (2006). Pharmacokinetics of tiletamine and zolazepam (Telazol^®^) in anesthetized pigs. J. Vet. Pharmacol. Ther..

[31] Miyabe-Nishiwaki T., Miwa M., Konoike N., Kaneko A., Ishigami A., Natsume T., MacIntosh A.J.J., Nakamura K. (2020). Evaluation of anaesthetic and cardiorespiratory effects after intramuscular administration of alfaxalone alone, alfaxalone-ketamine and alfaxalone-butorphanol-medetomidine in common marmosets (*Callithrix jacchus*). J. Med. Primatol..

[32] Bronson E., Deem S.L., Westermann L.C.P., Alpire S.A., Emmons L.H. (2021). Field Anesthesia of the Maned Wolf (*Chrysocyon brachyurus*) in Bolivia. J. Wildl. Dis..

[33] Coppola F., D’Addio E., Casini L., Sagona S., Felicioli A. (2020). Field Chemical Immobilization of Free-Ranging Crested Porcupines with Zoletil^®^: A Reviewed Dosage. Vet. Sci..

