# Supplementary material for: Evaluation of Anesthetic and Cardiorespiratory Effects after Intramuscular Administration of Three Different Doses of Telazol® in Common Marmosets (Callithrix jacchus)
Source: Vet Sci. 2023 Feb 3;10(2):116. doi: 10.3390/vetsci10020116 (PMC9959354; doi:10.3390/vetsci10020116)
Supplement: Supplementary file 1 [file vetsci-10-00116-s001.zip › vetsci-2020414-Table S1 Median and standard deviation of physiological data collected during first 45 minutes of sedation or anesthesia events.pdf]

Heart Rate (SPO2 monitor)

Dose	5 mg/kg		10 mg/kg		15 mg/kg	
Minutes	Median	SD	Median	SD	Median	SD
5	347.5	91.65	341	58.95	310	57.52
15	393.5	52.63	229	50.53	231.5	51.33
30	228.5	65.8	214	43.68	221.5	78.57
45			225	63.23	229	41.02

SPO2

Dose	5 mg/kg		10 mg/kg		15 mg/kg	
Minutes	Median	SD	Median	SD	Median	SD
5	85	5.45	86	7.28	88	4.6
15	90.5	5.81	88.5	6.49	85	4.78
30	90	3.27	90	4.8	88	6.83
45				3.11	88.5	4.9

Respiration Rate

Dose	5 mg/kg		10 mg/kg		15 mg/kg	
Minutes	Median	SD	Median	SD	Median	SD
5	92	225.7	76	29.4	74	27.34
15	64	24.37	62	15.69	58	28.35
30	60	14.24	54	16.55	66	32.28
45			64	19.78	56	19.42

Heart Rate (Blood pressure monitor)

Dose	5 mg/kg		10 mg/kg		15 mg/kg	
Minutes	Median	SD	Median	SD	Median	SD
5	298.5	87.64	305.5	73.56	265.5	61.49
15	253	49.77	225	46.99	209.5	53.44
30	240.5	70.84	192.5	49.64	204.5	71.96
45			225.5	58.14	229.5	36.04

Blood Pressure Systolic

Dose	5 mg/kg		10 mg/kg		15 mg/kg	
Minutes	Median	SD	Median	SD	Median	SD
5	127.45	28	105.8	21.11	109.95	20.35
15	107.8	13.48	100.65	14.53	103.3	17.12
30	111.95	24	100.8	10.7	111	19.39
45			114.95	10.2	117.15	13.98

Blood Pressure Diastolic

Dose	5 mg/kg		10 mg/kg		15 mg/kg	
Minutes	Median	SD	Median	SD	Median	SD
5	57.45	12.25	54.8	7.71	52.15	10.28
15	58.95	10.65	51	7.8	46.1	5.6
30	56.6	12.58	51.1	7.18	51.6	11.41
45			59	5.14	53.3	7.76

Blood Pressure Mean

Dose	5 mg/kg		10 mg/kg		15 mg/kg	
Minutes	Median	SD	Median	SD	Median	SD
5	92.15	14.32	79.83	13.85	80.66	15.09
15	84.4	11.58	75.49	10.56	74.5	9.77
30	84.5	17.75	76	8.33	81.06	13.74
45			84.91	6.94	84.87	10.29
