# Supplementary figures and images for: Evaluation of Anesthetic and Cardiorespiratory Effects after Intramuscular Administration of Three Different Doses of Telazol® in Common Marmosets (Callithrix jacchus)
Source: Vet Sci. 2023 Feb 3;10(2):116. doi: 10.3390/vetsci10020116 (PMC9959354; doi:10.3390/vetsci10020116)

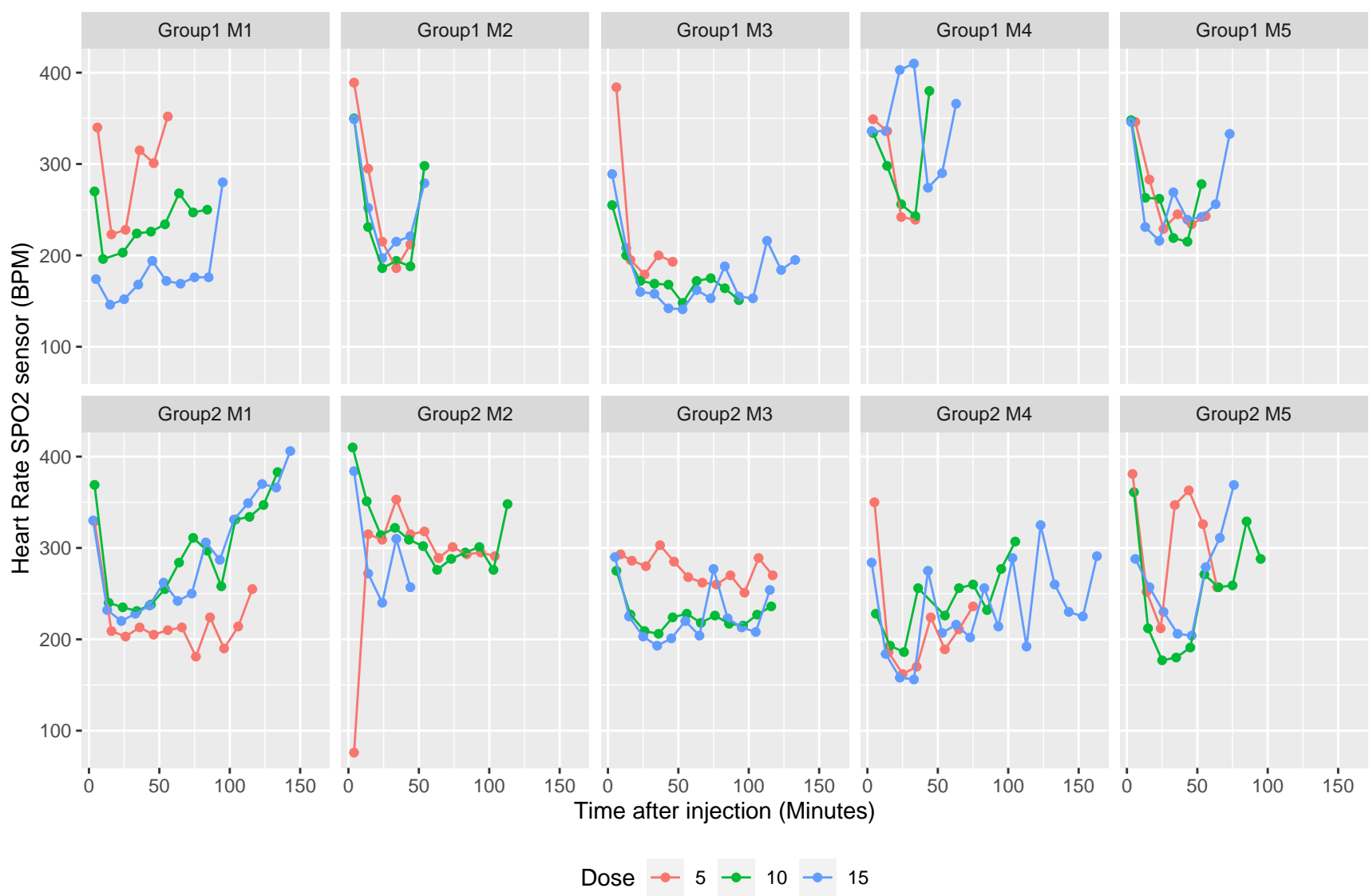

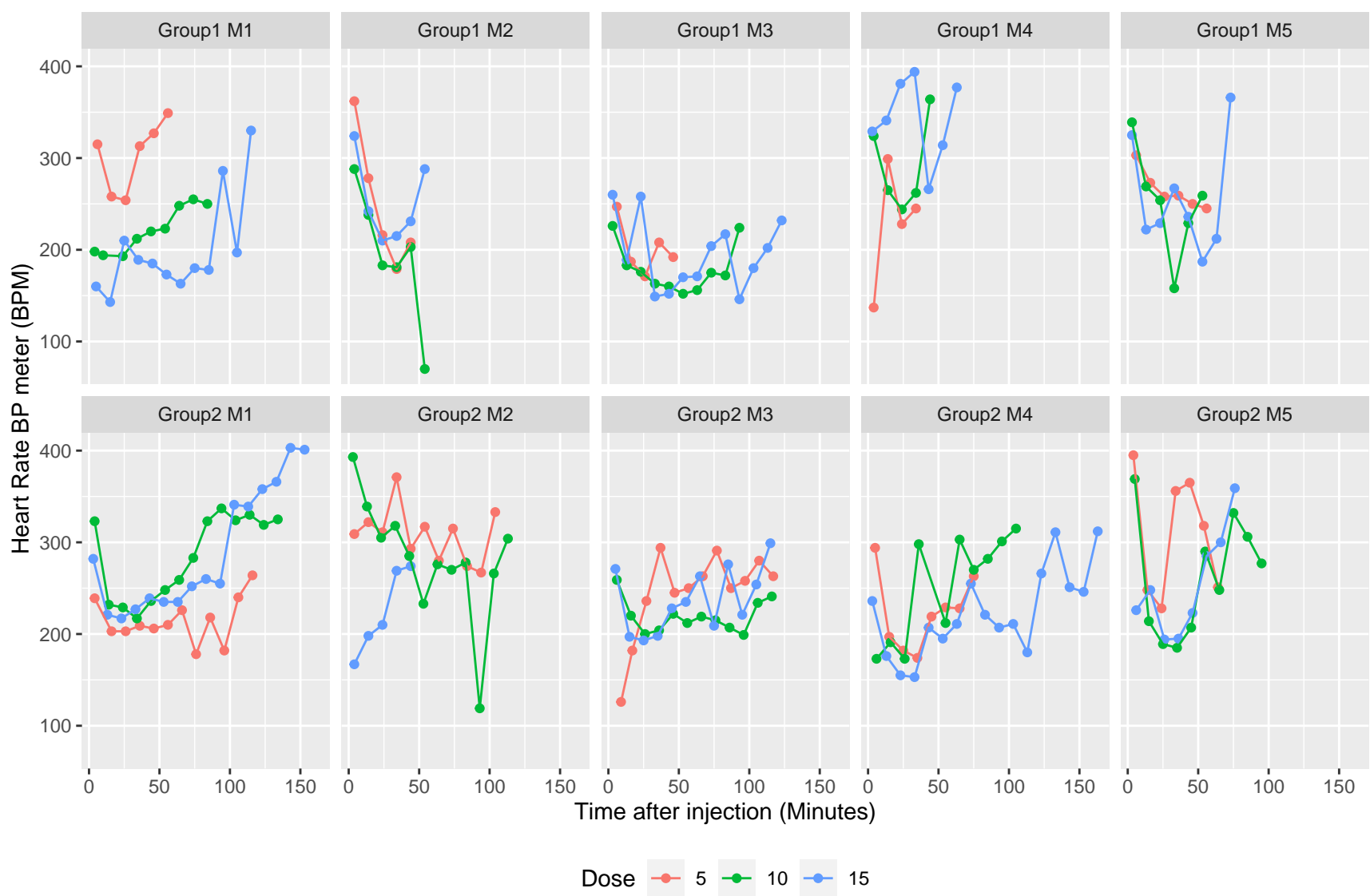

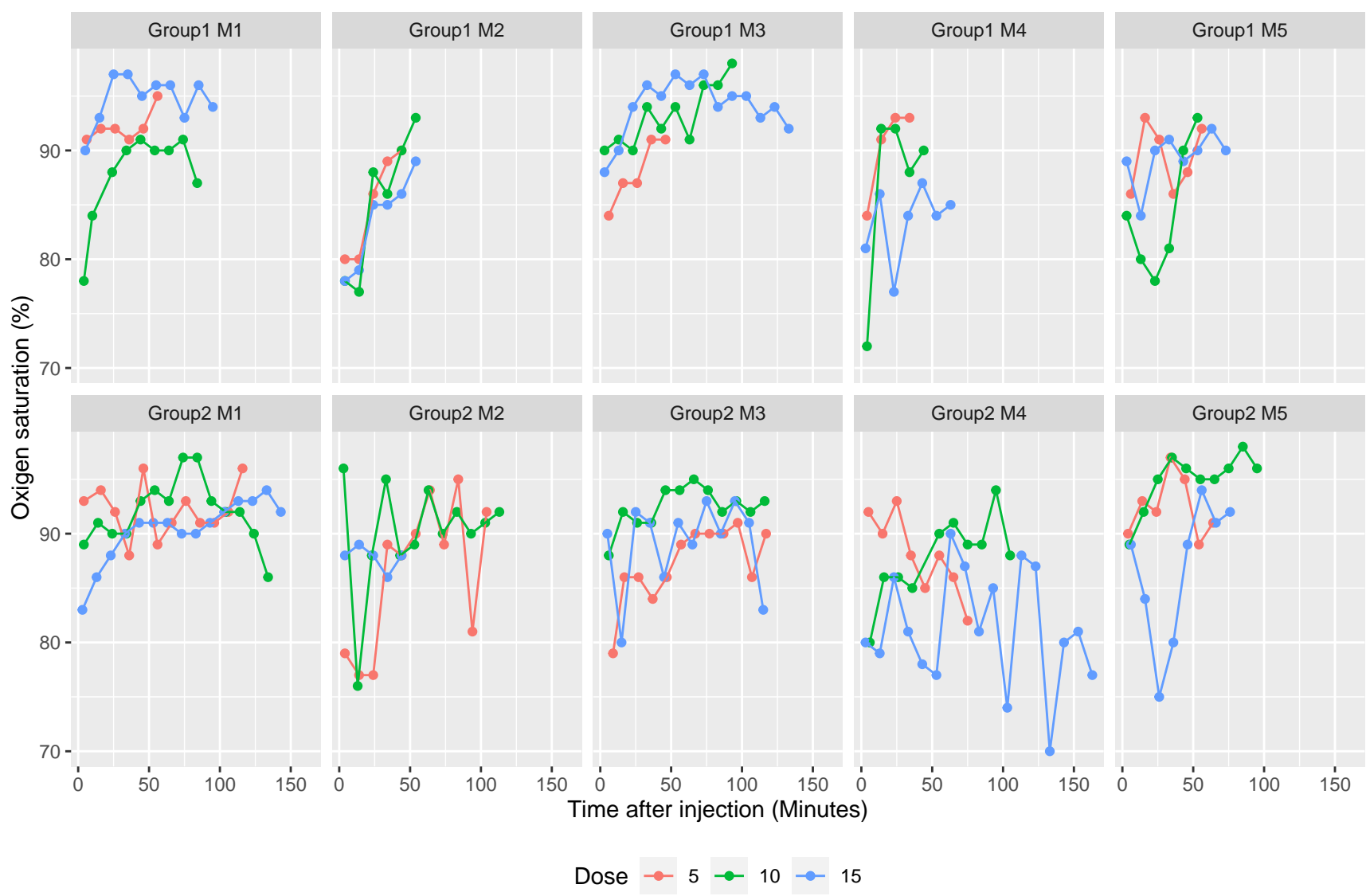

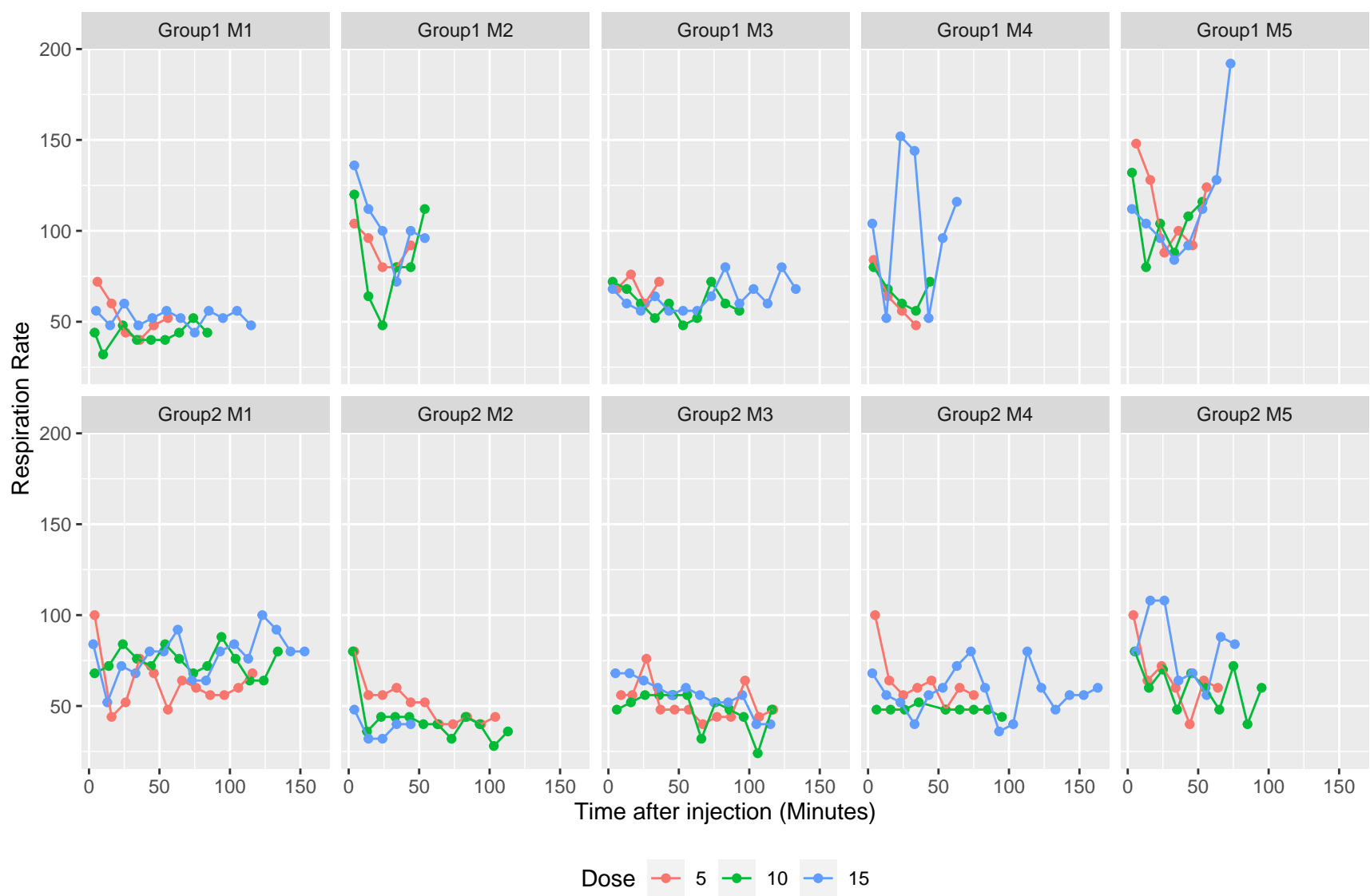

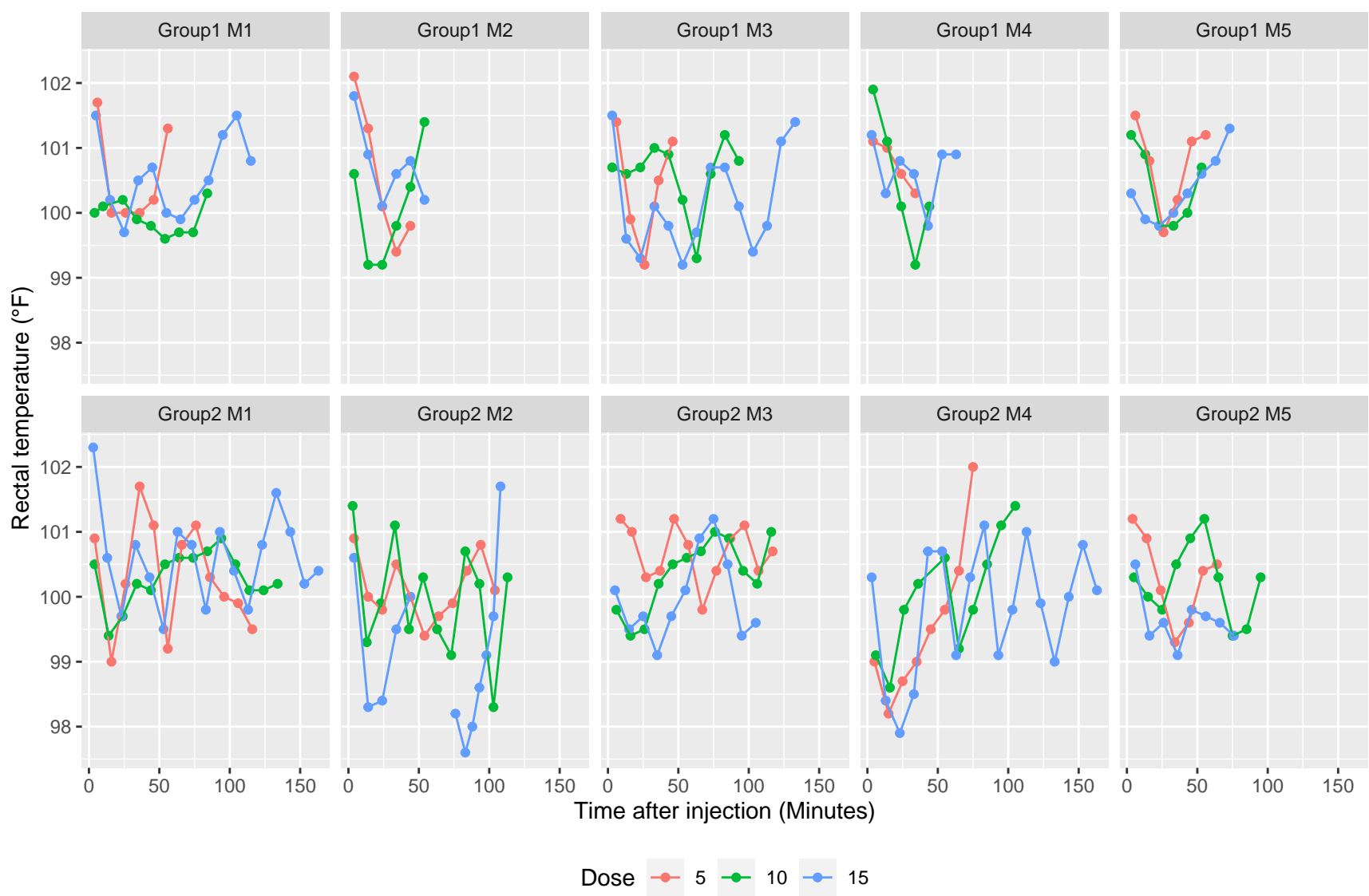

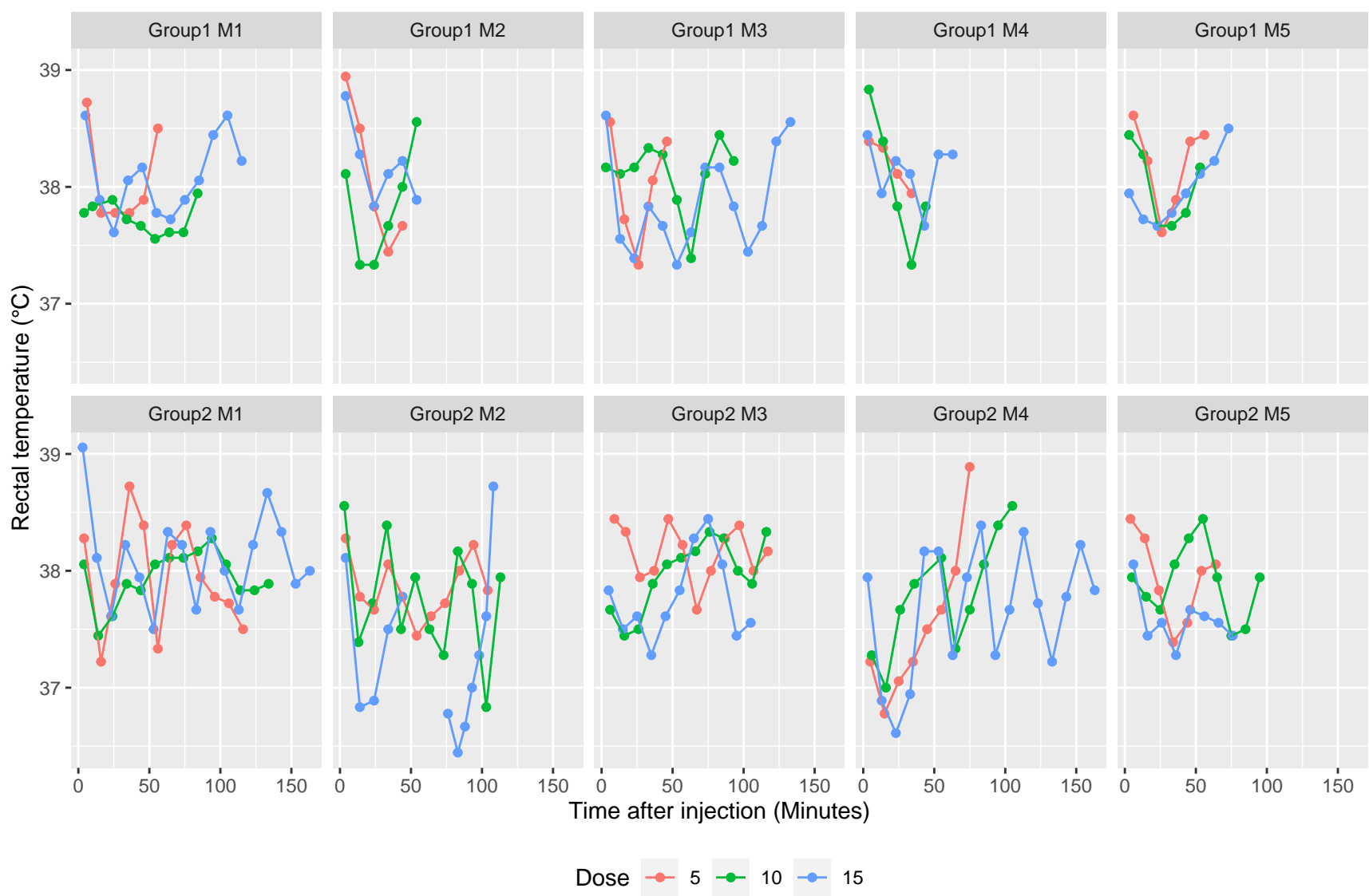

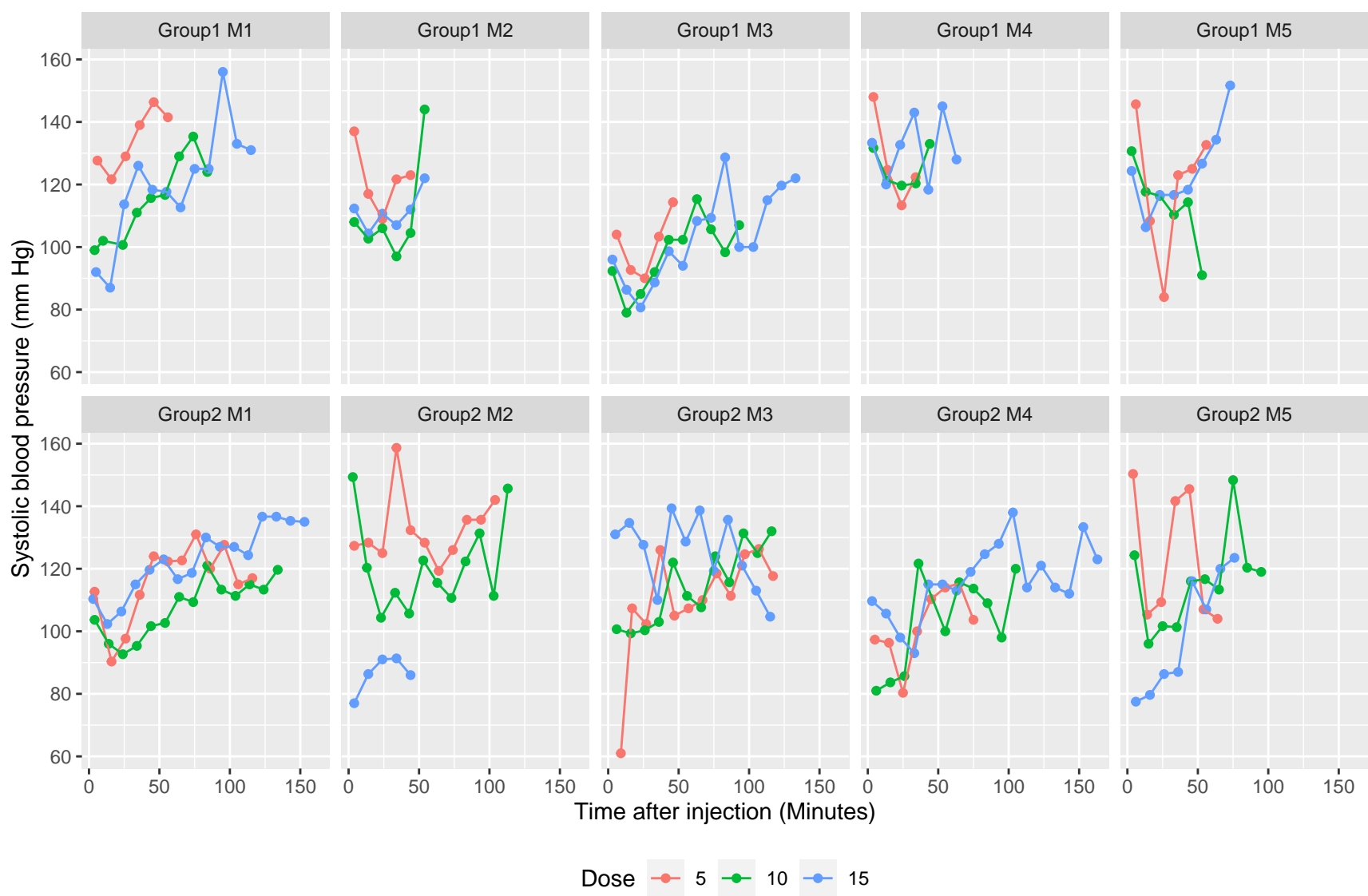

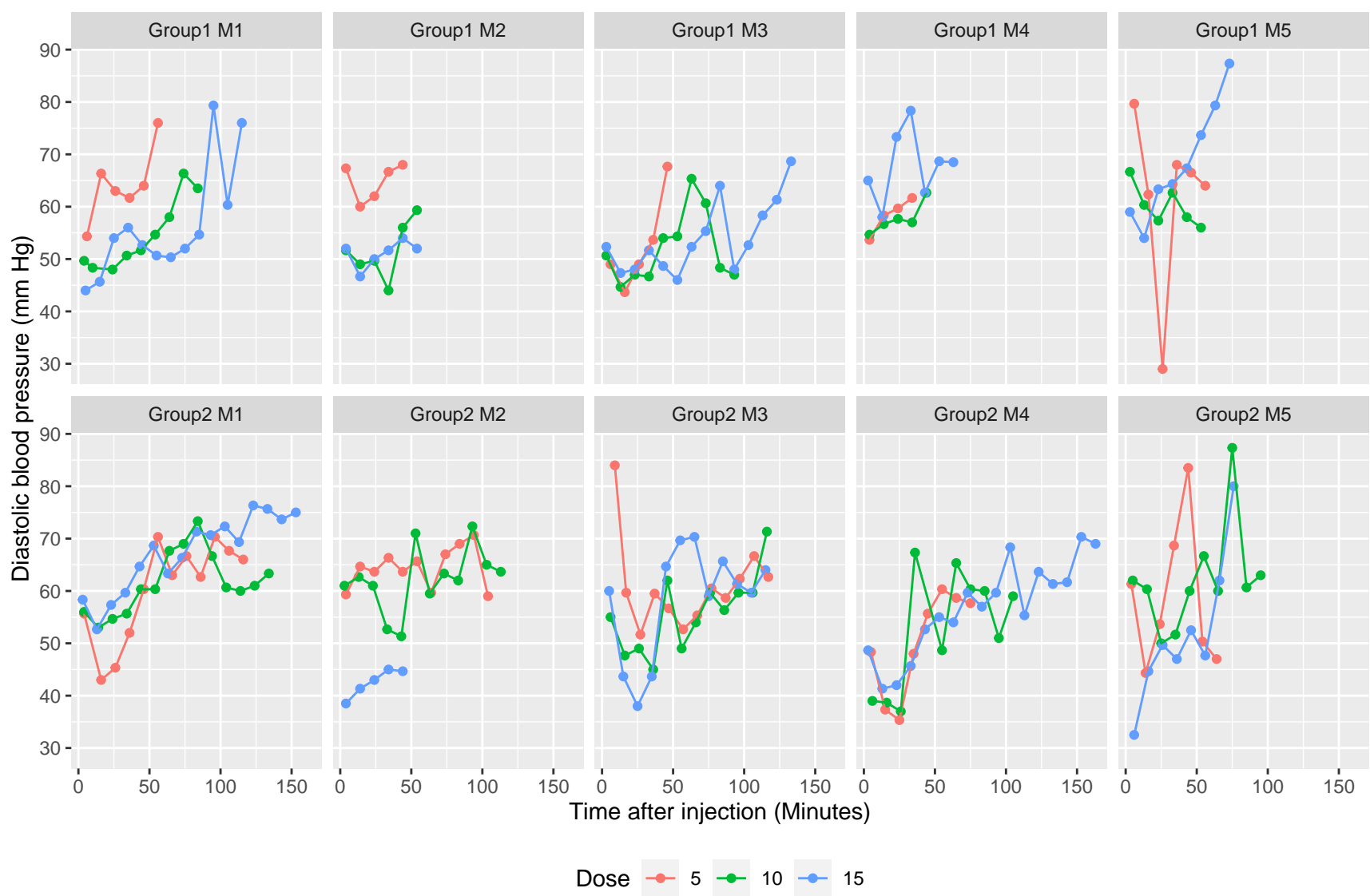

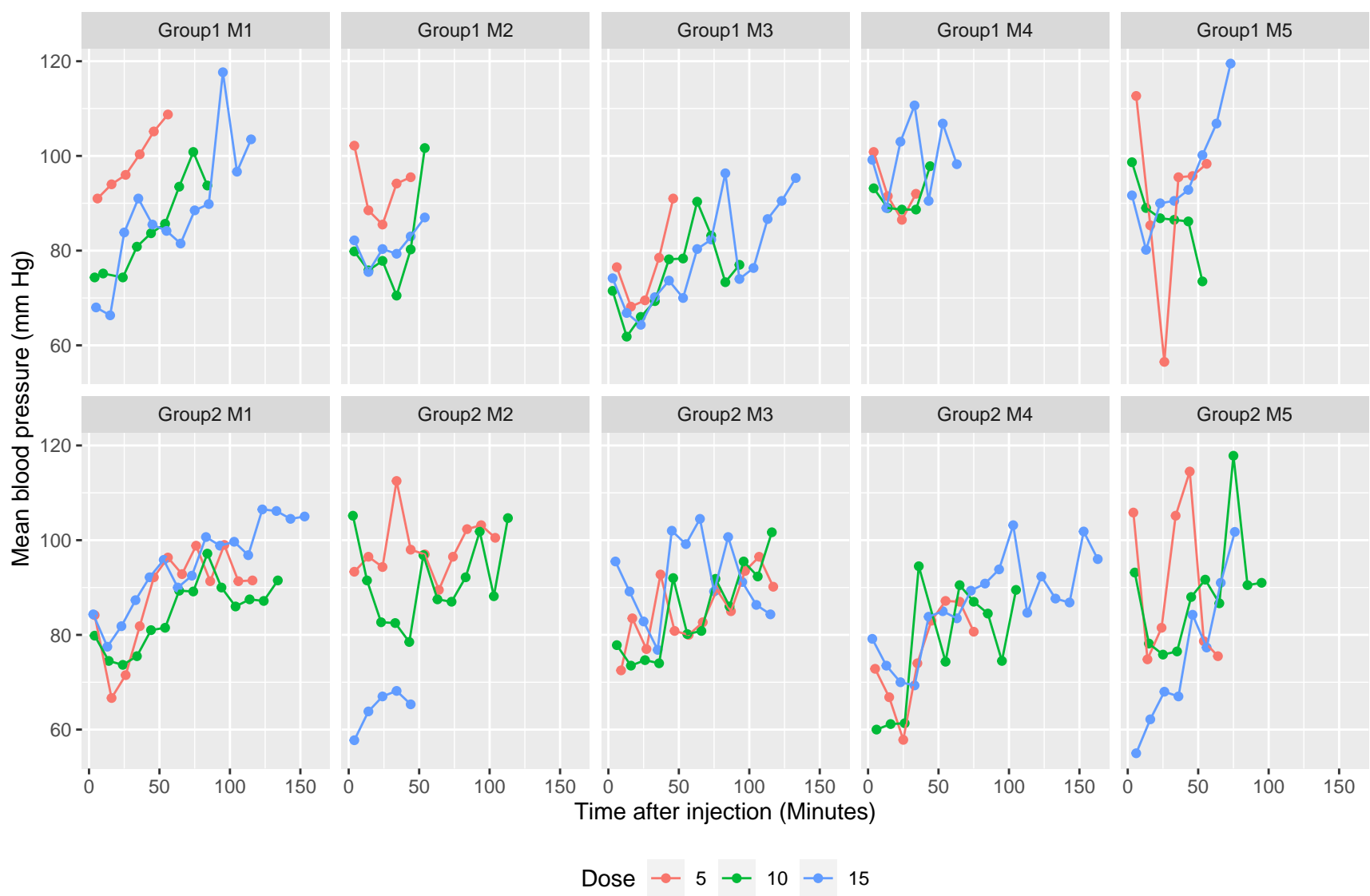

Supplement: Supplementary file 1 [file vetsci-10-00116-s001.zip › vetsci-2020414-Figure S1 The results of the objective scoring for each assessment used to determine depth of the sedation or anesthesia event .pdf]
